# Addition *Pinus massoniana* fallen wood improved the growth of *Plagiomnium acutum* in a substrate cultivation

**DOI:** 10.1038/s41598-022-21901-1

**Published:** 2022-10-22

**Authors:** Bingyang Shi, Xiurong Wang, Shuoyuan Yang, Hongmei Chen, Yang Zhao

**Affiliations:** Forestry College, Guizhou University, Huaxi District, Guiyang, 550025 Guizhou China

**Keywords:** Plant sciences, Plant breeding, Plant physiology, Plant reproduction

## Abstract

Soilless culture has been widely used in horticultural plant production, but little research has been done on bryophyte. In this study, we selected a cultivation substrate mixed and proportioned with garden soil, granular soil, grass charcoal soil, general-purpose nutrient soil, and decomposed grade II, III, and IV fallen wood of *Pinus massoniana* as the raw materials of soilless substrate to investigate its effects on the growth and physiology of *Plagiomnium acutum*. The results showed that the total porosity, water-holding porosity, and water-holding capacity of the mixed substrate containing fallen wood of *P. massoniana* were significantly higher than those of other cultivated substrates. The average cover of the *P. acutum* was significantly and positively correlated with the substrate’s total porosity and water-holding porosity. Chlorophyll content was highly significantly and positively correlated with the water holding capacity and total nitrogen content of the substrate. Among them, V_III decomposition grade Pinus massoniana fallen log_:V_grass charcoal soil_ = 1:1 (SW8) substrate had the highest overall evaluation index and the best overall growth condition of *P. acutum*. In summary, V_III decomposition grade Pinus massoniana fallen log_:V_grass charcoal soil_ = 1:1 (SW8) substrate can be the best substrate for cultivation of *P. acutum*. The addition of *P. massoniana* fallen wood to the soil substrate increased the total porosity, water-holding porosity, and water-holding capacity of the substrate, which was conducive to the growth of *P. acutum* and the increase of chlorophyll content.

## Introduction

Soilless growing systems were characterized by dense plants roots confined in a small volume of growth medium, as well as high rates of metabolic activity, respiration and growth^1^. In recent years, soilless culture had been widely used in vegetable and flower production. Compared to soil cultivation, the physicochemical properties of soilless substrates were more homogeneous, had low pH buffering capacity, saving water and nutrients and reducing the waste of resources^2^. Soilless culture was mainly divided into three types: substrate culture, hydroponics, and aerosol culture, among which substrate culture was the most widely used in practical production^3^. According to the different substrate, cultivation could be divided into three types: inorganic substrate cultivation, organic substrate cultivation, and mixed substrate cultivation. Among them, the mixed substrate was made by mixing materials with different structure and properties, and the advantages of physical and chemical properties could be complemented between each material component, which made the mixed substrate better than inorganic and organic substrate in terms of water, gas and fertilizer coordination^4^. Soilless culture technology had many advantages, but there were fewer studies of their application in the cultivation of bryophytes. Selecting a new high-quality cultivation substrate suitable for the growth of bryophytes was the key to the industrial cultivation of bryophytes.

In addition to the influence of substrate, the ambient temperature, humidity and light also affected the growth of moss. The strong light could change the growth and morphology of bryophytes, and excessive or insufficient water could also lead to physiological changes of bryophytes, thus affecting the normal growth of bryophytes^5^. Different bryophytes had different environmental requirements. For example, research of Ji et al. showed that compared with *Thuidium cymbifolium*, the *Mnium immarginatum* were more resistant to negative^6^; It was found that the optimal water supply for *Hypnum plumaeforme*, *Plagiomnium acutum*, and *Leucobryum juniperoideum* were different^7^; The photosynthesis of *Hypnum plumaeforme* and *Leucobryum juniperoideum* was the strongest when the light transmittance was 30.52%, the chlorophyll content of *Leucobryum juniperoideum* was the highest at the light transmittance of 1.82%, and the negative tolerance was the strongest^8^. It could be seen that different species of bryophytes had different environmental requirements, so it is necessary to consider the suitable environmental factors for their growth in bryophyte culture.

*Pseudocorythion acutum* has long branches and strong covering ability, and beautiful appearance, so it was often used in garden landscaping, bonsai, micro landscape, and as moss jade ball. It has hemostatic function^9^ and was also a common plant material for making ecological tanks, which meant high medicinal value and market application value. At present, there were few studies on the propagation and cultivation of *P. acutum*, and the huge market demand has led to severe harvesting of *P. acutum* in the wild, which has caused severe damage to the natural ecological environment. Therefore, rapid and effective propagation and cultivation technique is a critical way to address the market demand of *P. acutum*.

*Pinus massoniana* was a significant forest species and occupies first place in terms of forest area and growing stock volume in Guizhou Province. With the natural regeneration and succession of forests, there were a large number of *P. massoniana* fallen wood and abundant epiphytic bryophytes growing on them. Bryophytes had high water storage capacity^10^ and nitrogen fixation capacity, which could promote the decomposition of fallen wood and increased the availability of soil nitrogen, and was conducive to the nutrient cycling and growth of forest. In recent years, a large number of studies had proved that bryophytes played an important role in forest regeneration and nutrient cycling^11,12^ and grow better on fallen forest wood^13,14^. The degree of decaying wood affected the life shape and gametophyte growth of bryophytes^15,16^. It could be seen that bryophytes interact with forest fallen wood in a mutualistic way, but what is the mechanism by which forest fallen wood affect the growth of bryophytes? It is urgent to study whether it can be used as a new excellent culture substrate to cultivate moss.

Based on this, this study selected the present high market application value, *P. acutum*, and mixures of four kinds of soils and different decomposition grades of fallen *P. massoniana* as the substrate material for cultivation experiments to evaluate whether the mixed substrate of *P. massoniana* fallen wood could be used as a new soilless culture substrate to promote the growth of *P. acutum*. By studying the formulation of suitable substrate and related influencing factors, we aim to provide theoretical basis and technical support for the propagation and cultivation of *P. acutum*.

## Results

### Analysis of physical and chemical properties of different cultivation substrates

**Figure 1:**
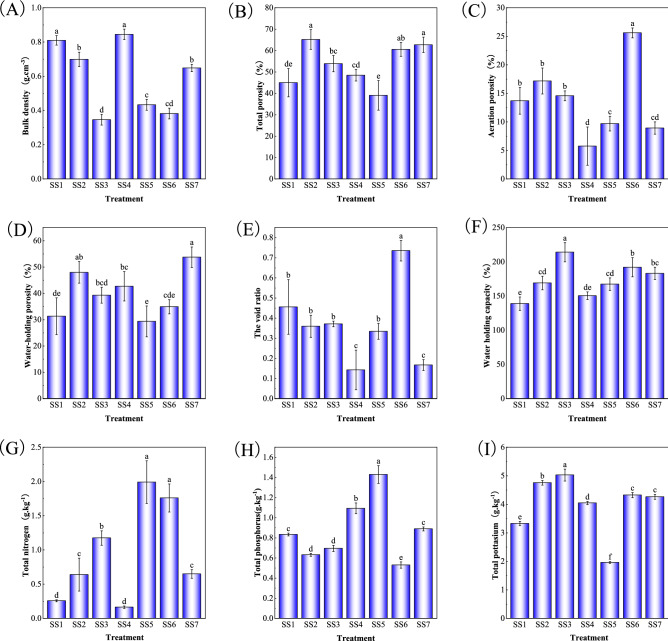
Basic physicochemical properties of soil–soil mixed substrates. Means in columns followed by different lowercase letters are significantly different at P < 0.05.

**Figure 2:**
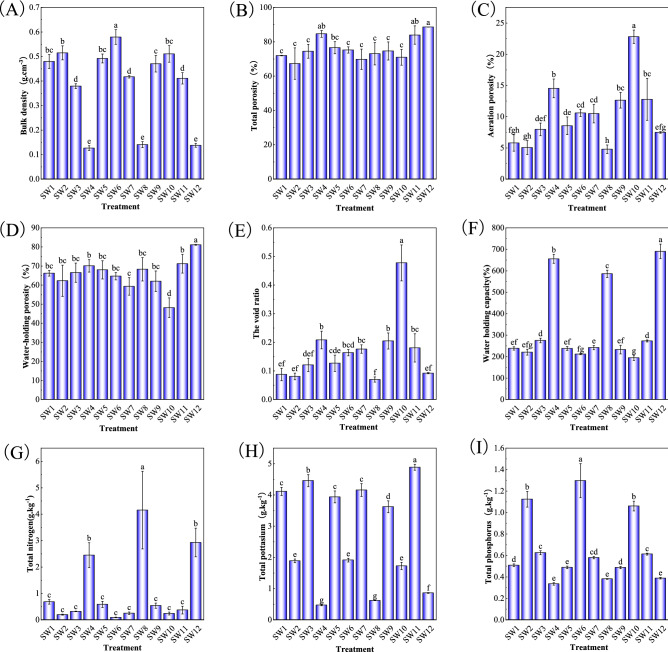
Basic physicochemical properties of soil–wood mixed substrates. Means in columns followed by different lowercase letters are significantly different at P < 0.05.

From Figs. 1 and 2, it can be seen that the total porosity, water holding porosity and water holding capacity of soil–wood mixture substrate (SW) were significantly increased after the fallen wood was added into the substrate, while the bulk density of SW decreased. Treatments with charcoal soil had significantly more total nitrogen, while with granular soil had more total phosphorus, and with garden soil or general nutrient soil component had more total potassium in the substrates.

### Effects of different substrate treatments and environmental factors on the growth and morphological indexes of *P. acutum*

**Figure 3:**
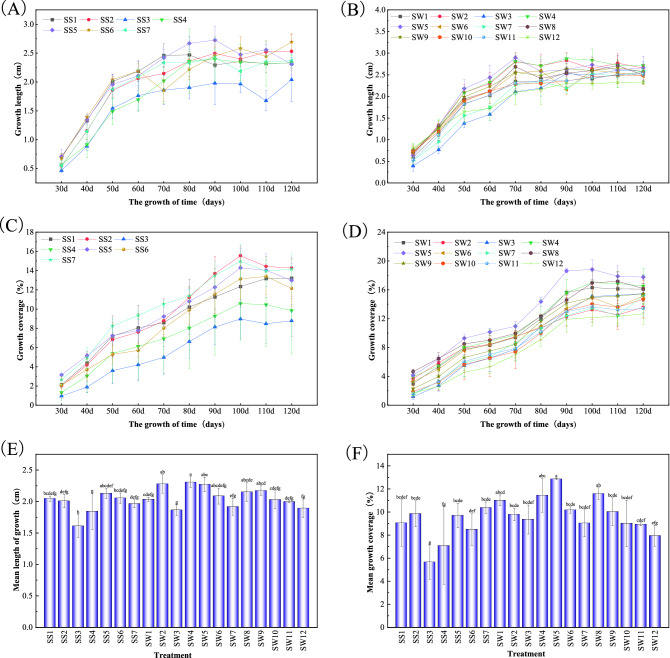
Different substrate treatments on the growth and morphological indices of Plagiomnium acutum,including growth length (**A,B**), coverage (**C,D**), mean growth length (**E**), mean coverage (**F**). Means in columns followed by different lowercase letters are significantly different at P < 0.05.

There were significant differences in the length and coverage under different mixed substrates in different periods. As shown in Fig. 3A,B, at 30 days of cultivation, the length of *P. acutum* grown on the SW12 substrate was the longest, 0.80 cm, while the length on SW3 substrate was the shortest, 0.39 cm. The maximum length reached 2.87 cm among all treatments at 90 days under SW4 substrate treatment. From Fig. 3C,D, the coverage under SW8 substrate was the highest (4.67%), while that under SS3 substrate was the lowest (0.96%). During the whole experiment period, the maximum coverage reached 18.83% at 100 days under SW5 treatment in all treatments.

To precisely determine the differences in the growth conditions of *P. acutum* in different cultivation substrates, the average length and coverage under different treatments were taken for each period within 120 days. In Fig. 3E,F, among all treatments SW4 had the largest mean growth length, which was significantly different from all other treatments except for SW2, SW5, SW6, SW8, SW9 and SS5. The mean cover of *P. acutum* under SW5 treatment was the largest and significantly different from all other treatments except SW4, SW1, and SW8. The average length and coverage of SS3 were the smallest. In summary, the growth condition of *P. acutum* was better under SW4, SW5 and SW8 treatments.

In addition, the changes of ambient temperature and humidity also had effects on the growth of bryophytes. The growth rate of *P. acutum* was the fastest during 30–50 days and slowed down during 50–60 days, which might be because the air humidity was higher in 30–50 days than that in 50–60 days (Figs. 3, 8). During the period of 70 to 80 days, the temperature rose, the air humidity dropped sharply, and the length of *P. acutum* changed significantly or even showed negative growth, which might be due to the leaf shrinkage of *P. acutum* in response to the high-temperature environment, and led to the measurement error. The coverage of *P. acutum* was in the growth stage from 30 to 90 days, but decreased from 90 to 120 days, possibly because the temperature rose and the air humidity decreased significantly during the period, which affected the growth and spread of *P. acutum* (Figs. 3, 8). In conclusion, temperature and humidity played an important role in the growth of *P. acutum*, and a humid environment might be more suitable for its growth.

### Effects of different substrate treatments on some physiological indexes of *P. acutum*

**Figure 4:**
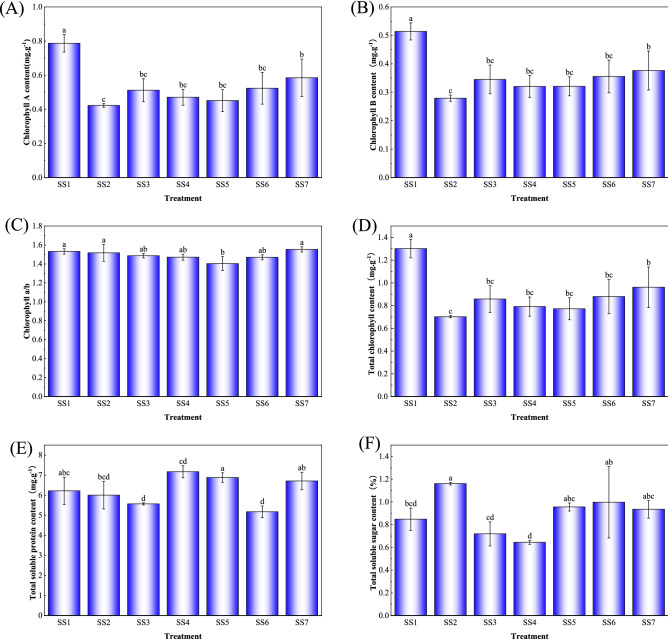
Effect of soil–soil mixed substrate treatment on some physiological indexes of *P. acutum.* Means in columns followed by different lowercase letters are significantly different at P < 0.05.

**Figure 5:**
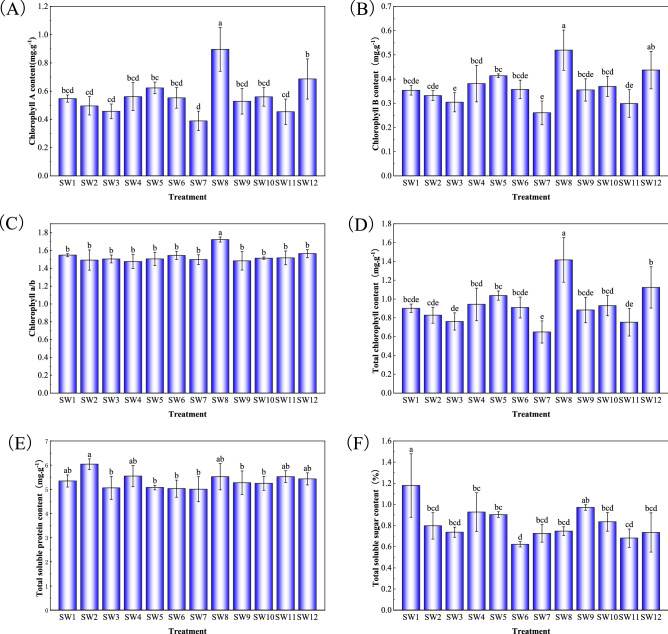
Effect of soil–wood mixed substrate treatment on some physiological indexes of *P. acutum.* Means in columns followed by different lowercase letters are significantly different at P < 0.05.

Different mixed substrates had significant effects on some physiological indexes of *P. acutum* (Figs. 4, 5). Chlorophyll could reflect the photosynthetic ability of plants^17^, and photosynthesis of appropriate intensity was conducive to the accumulation of organic matter in plants^18^. The content of chlorophyll a, chlorophyll b, and total chlorophyll of *P. acutum* in SS1 was the highest and significantly different from the other soil–soil mixed substrate treatment. In the soil–wood mixed substrate treatment, the chlorophyll a, chlorophyll b, chlorophyll a/b value, and total chlorophyll content of *P. acutum* with SW8 were the most abundant, while those in SW7 were the least, which showed that the growth and metabolic capacity of *P. acutum* under SW8 was the most robust performance among all treatments. Soluble sugars provided most of the energy needed by plant growth, and soluble protein content reflected the level of enzyme activity in the plant. In higher plants, soluble sugars and soluble proteins maintained the osmotic balance of the plant body cells and improve plant resistance^19^. Soluble sugars and soluble protein content contributed to the improvement of plants resistance. Among all the treatments, the soluble sugar content of *P. acutum* in SW1 treatment was the highest, and SS2 was second only to SW1. The soluble protein content in SS5 was the highest. Those showed that SW1 and SS5 substrate treatments could increase the growth resistance of *P. acutum*.

### Effects of different cultivation substrate treatments on the growth and development of the *P. acutum*

**Figure 6:**
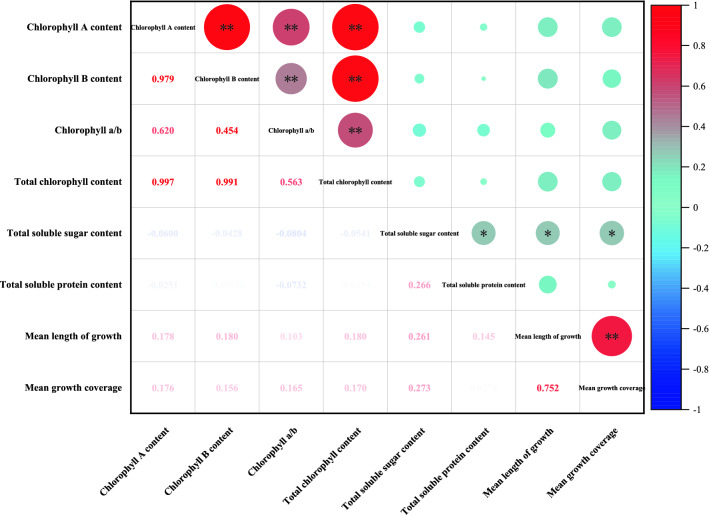
Pearson correlation analysis among the growth and physiological indexes of *P. acutum*. **Indicates extremely significant correlation at the P < 0.01 level, *indicates significant correlation at the P < 0.05 level.

Pearson’s correlation analysis among the physiological growth indicators of *P. acutum* showed that there was a highly significant positive correlation between chlorophyll a, chlorophyll b, chlorophyll a/b values and total chlorophyll content of *P. acutum*. The total soluble protein content was significantly positively correlated with the total soluble sugar content, and the average length was also significantly positively related to the total soluble sugar content of *P. acutum* (Fig. 6). The average length and coverage of the moss were proportional to its physiological indexes, which could show the growth and development of the moss (Table 1).

**Table 1:** Comprehensive evaluation of the growth and development of *P. acutum* with different cultivation substrate treatments.

Treatment	Each indicator belongs to the function value								Comprehensive evaluation index	Rank
	Chlorophyll A content	Chlorophyll B content	Chlorophyll a/b	Total chlorophyll content	Total soluble protein content	Total soluble sugar content	Mean length of growth	Mean coverage		
SS1	0.7849	0.9785	0.4011	0.8503	0.6460	0.4041	0.6268	0.4722	0.6455	2
SS2	0.0664	0.0724	0.3562	0.0683	0.5318	0.9653	0.5694	0.5835	0.4017	11
SS3	0.2427	0.3270	0.2601	0.2713	0.1787	0.1728	0.0000	0.0000	0.1816	19
SS4	0.1610	0.2317	0.2141	0.1849	0.2586	0.0395	0.3349	0.1994	0.2030	17
SS5	0.1227	0.2336	0.0000	0.1603	1.0000	0.5976	0.7464	0.5612	0.4277	8
SS6	0.2655	0.3676	0.2096	0.3001	0.0909	0.6725	0.6364	0.3942	0.3671	13
SS7	0.3859	0.4473	0.4703	0.4067	0.9084	0.5602	0.5072	0.6549	0.5426	4
SW1	0.3116	0.3598	0.4529	0.3279	0.1808	1.0000	0.6077	0.7444	0.4981	6
SW2	0.2106	0.2747	0.2786	0.2323	0.5519	0.3136	0.9617	0.5742	0.4247	9
SW3	0.1341	0.1693	0.3177	0.1460	0.0275	0.2054	0.3636	0.5135	0.2346	16
SW4	0.3393	0.4668	0.2301	0.3823	0.2890	0.5488	1.0000	0.8052	0.5077	5
SW5	0.4608	0.5910	0.3194	0.5048	0.0366	0.5044	0.9474	1.0000	0.5456	3
SW6	0.3222	0.3729	0.4430	0.3393	0.0116	0.0000	0.6842	0.6285	0.3502	14
SW7	0.0000	0.0000	0.2963	0.0000	0.0000	0.1860	0.4685	0.4685	0.1739	18
SW8	1.0000	1.0000	1.0000	1.0000	0.2757	0.2235	0.8256	0.8256	0.7625	1
SW9	0.2743	0.3643	0.2538	0.3047	0.1431	0.6257	0.6071	0.6071	0.4227	10
SW10	0.3359	0.4210	0.3459	0.3647	0.1291	0.3820	0.4661	0.4661	0.3810	12
SW11	0.1278	0.1489	0.3576	0.1348	0.2758	0.1052	0.4559	0.4559	0.2701	15
SW12	0.5856	0.6819	0.5059	0.6182	0.2278	0.2016	0.3168	0.3168	0.4431	7

Chlorophyll content reflected the photosynthetic capacity of plants and could represent the growth status of plants to a certain extent. The indexes of length and coverage could reflect the scene formation rate of moss and were also the most important indexes to judge whether moss could achieve rapid propagation and cultivation. It could be seen from Table 1 that the top six treatments of the cultivation substrates suitable for the growth of mosses, they were SW8, SS1, SW5, SS7, SW4, and SW1. Combined with the membership function values and rankings of each index in Table 3, we found that the SS1 substrate could effectively improve the chlorophyll content of mosses, which was conducive to enhancing their photosynthetic capacity. Substrates SS7 and SW1 could increase soluble protein content and soluble sugar content, which was beneficial to enhance plant resistance. SW5 and SW4 substrates were conducive to the growth and spread of *P. acutum*, and increased the scene formation rate of *P. acutum*. However, on the SW8 substrate, the chlorophyll content, growth length, and coverage of *P. acutum* not only the growth status was better, but also the scene formation rate was faster. Therefore, considering the market background, the SW8 substrate could be used as the best substrate for the cultivation of *P. acutum.*

## Discussion

**Figure 7:**
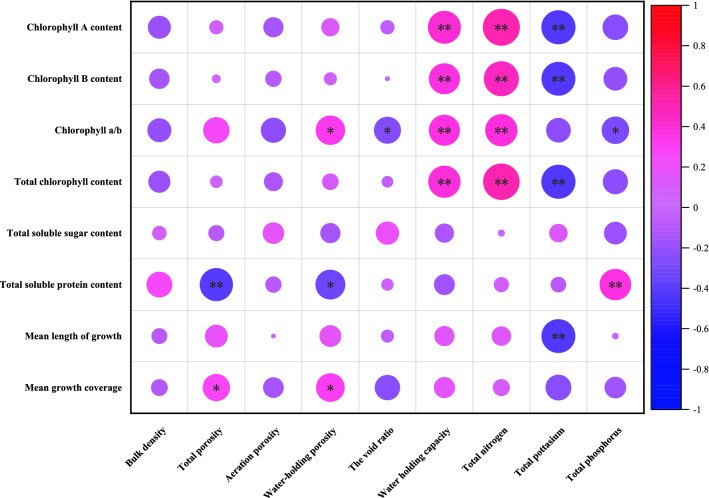
Pearson correlation analysis of cultivation substrate property indexes and physiological indexes of growth of *P. acutum*. **Indicates extremely significant correlation at the P < 0.01 level, *indicates significant correlation at the P < 0.05 level.

Plant morphological indicators could reflect the strength of plant growth and could be used to determine the differences in the effects of different cultivation substrates on plant growth, and judge the superiority or inferiority of that cultivation substrate^20^. The material, structure, and nature of the substrate were the key factors affecting the plant growth status^21^. In this study, the total porosity and water-holding porosity of the substrate were significantly positively correlated with the average coverage, and the water-holding capacity of the substrate was highly significantly and positively correlated with the chlorophyll content (Fig. 7). The bulk density of SW8, the best treatment for the growth of moss, was 0.14 g/cm^3^, the total porosity was 73.08%, and the void ratio was 0.07, of which except the total porosity, the other two indicator were lower than the Industry Standard for Floriculture Substrates (LY/T2700-2016) (in which the three indicators were 0.30–1.80 g/cm^3^, 50–95%, and 0.25–0.67)^22^. Some studies also pointed out that good substrate structure ultilized fixation and growth of plant roots^23^. Because the moss was small in size and light in weight and difficult to collapse, this indicated the Industry Standard for Floriculture Substrates (LY/T2700-2016) needs to be improved for moss cultivation. Comparing the soil–wood mixed substrate (SW) treatment and the soil–soil mixed substrate (SS) treatment, it was found that the overall growth of *P. acutum* was better under the soil–wood mixed substrate (SW) treatment, so using the fallen wood could increase the porosity and water-holding capacity of the substrate and reduce the substrate capacity, which would be beneficial to the growth and spread of *P. acutum*.

Nitrogen is an essential component of plant protein production and plays a vital role in the process of photosynthesis^24^. In this study, the chlorophyll content of *P. acutum* was highly significantly and positively correlated with the total nitrogen content, and the soluble protein content was significantly positively correlated with the total phosphorus content of the substrate (Fig. 7). This indicated that substrate nitrogen content had no significant effect on the growth of *P. acutum*, while had a certain effect on leaf color, and phosphorus content was beneficial to improve the resilience of the moss, which was similar to the findings of Tahir et al.^25^ and Yang et al.^26^.

In addition, the changes in temperature and humidity during cultivation also had important effects on plant growth^27^. In this study, *P. acutum* was also sensitive to changes in temperature and humidity. High temperature and dry environment were not conductive to the growth of *P. acutum*, and may even have an inhibitory effect and lead to its death, which was similar to the research results of Liu et al.^28^. Secondly, we found that when the air humidity increased, the growth rate of *P. acutum* increased, indicated that *P. acutum* preferred humid environments, which was also consistent with the results of Li et al., who proved that *P. acutum* was more suitable for humid and cloudy environments^29^. The focus of this study was on the selection of cultivation substrate, and the most suitable temperature and humidity for the growth of the moss need to be further studied.

## Conclusions

Different cultivation substrates had specific effects on the growth and physiological traits of *P. acutum*. The substrate containing *P. massoniana* fallen wood had higher total porosity, water-holding porosity and water-holding capacity, which could effectively improve the reproduction and growth efficiency of *P. acutum*. The nutrient composition of the substrate had an effect on the leaf color traits and stress resistance of *P. acutum*. And the moist environment may be more suitable for the growth of *P. acutum*. Combined with the membership function values and the final ranking of each index, we found that the SS1 substrate could effectively improve the chlorophyll content of *P. acutum*, which was beneficial to enhance its photosynthetic capacity. SS7 and SW1 substrates could increase soluble protein content and soluble sugar content, which was beneficial to enhance plant resistance. SW5 and SW4 substrates could increase the scene formation rate of *P. acutum.* The chlorophyll content, length, and coverage of moss on SW8 substrate were outstanding, and not only the growth status was better, but also the scene formation rate was faster. Therefore, considering the factors of market background, V_IIIdecomposition grade_ _*Pinus massoniana*_ _fallen log_:V_grass charcoal soil=1:1_ (SW8) substrate could be used as the best substrate for cultivation of moss.

As a scarce ornamental plant in the market, the urgent problem need to be solved was the effective and rapid propagation and cultivation technology of *P. acutum*. The substrate formulation in this study has a high market development potential as a moss breeding substrate, and also provides a new model for soilless culture of bryophytes.

## Materials and methods

### Experimental site

**Figure 8:**
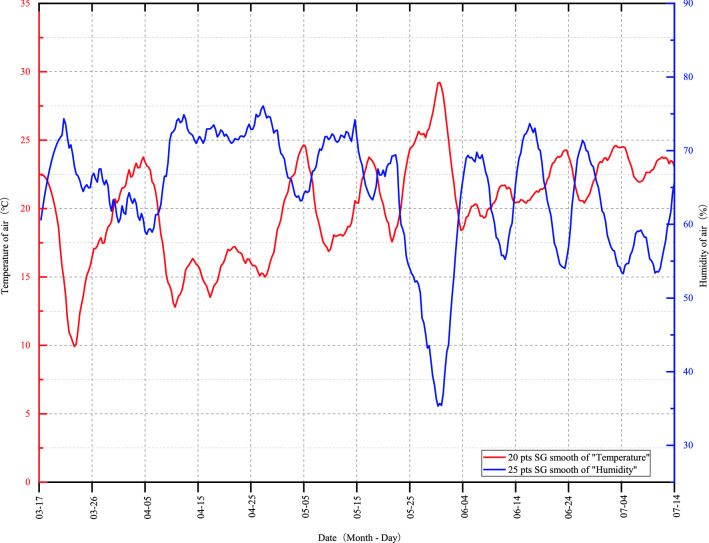
Dynamic change of air temperature and humidity during the experimental period (March 2021–July 2021).

The experiment was carried out from March to July 2021 in West Campus of Guizhou University located at Guiyang, Guizhou Province, China (106° 65′ E, 27° 22′ N), which has a typical subtropical, humid and mild climate, with plateau and monsoonal characteristics. The mean annual precipitation, annual average temperature, annual average relative humidity and annual average sunshine hours in Guiyang were 1129.5 mm, 15.3 ℃, 77%, and 1148.3 h respectively. Temperature and humidity changes were recorded with a thermohygrometer at 8:00–9:00, 12:00–13:00, and 17:00–18:00 each day during the experiment (Fig. 8). *P. acutum* prefered a shady and the suitable light range for it is 70% to 90% shading^30^, so the cultivation area were treated with three layers of shading net to ensure that the shading rate was above 70%. Spray and moisturize daily according to the actual situation.

### Cultivation substrate and plant material

Soil substrate include Granular soil, general-purpose nutrient soil, grass charcoal soil and garden soil. *P. massoniana* fallen wood was collected from the West Campus of Guizhou University, and divided into different decay grades (Table 2) regarding the classification and determination criteria of forest coarse woody residue decay grades by previous studies^13,31,32^. The mosses were harvested from Guanshan Lake Park in Guiyang City, Guizhou Province.

**Table 2:** Fallen log classification table.

Decision criteria	The decomposing level		
	II	III	IV
Needle	Absent	Absent	Absent
Bark	Partial shedding	Partly present only on large branches	Absent
Branches and twig	Branches present	Large branches present	Only large branches stub present
The trunk shape	Round	Round	Round to oval
The indirect method	Cambium decayed, knife blade penetrates 3 mm to 1 cm	Knife blade penetrates less than 2 cm	Severely decay, knife penetrates 2–5 cm
Structural integrity	Sapwood rotten heartwood intact	Sapwood missing heartwood intact	The heartwood has rotted
Wooden	Solid	Semi solid	Part of the tender
Wooden color	Primary colors	Primary color to fade	Primary color to fade

### Experimental design

The soils were disinfected by exposure to sunlight for 7 days and the bark, sapwood and heartwood of the fallen woods were sampled and then crushed and mixed separately before planting. Two groups of treatments were set up in the cultivation trials (Table 3), namely the soil–soil mixed substrate equal volume mixed homogeneous (SS) treatment and the soil–wood mixed substrate equal volume mixed homogeneous (SW) treatment. After laying the nonwoven fabric in the planting pots, put the same volume of mixed substrate in the pots and watered thoroughly before transplanting. The moss cultivation was done by gametophyte cultivation. The cultivation method borrowed from the mixed seeding method (area method + weight method) of Sun^33^, that is, the moss crushing by a crusher was weighed, and then it was evenly laid flat on the substrate with 4 cm × 4 cm area, and each treatment was repeated three times.

**Table 3:** Cultivation substrate treatment.

Cultivation type	Treatment	Substrate ratio (V:V)
Soil–soil mixed substrate (SS)	SS1	The garden soil:granular soil (1:1)
	SS2	The garden soil:general-purpose nutrient soil (1:1)
	SS3	General-purpose nutrient soil:grass charcoal soil (1:1)
	SS4	General-purpose nutrient soil:granular soil (1:1)
	SS5	Granular soil:grass charcoal soil (1:1)
	SS6	The garden soil:grass charcoal soil (1:1)
	SS7	Grass charcoal soil:granular soil:general-purpose nutrient soil:the garden soil (1:1:1:1)
Soil–wood mixed substrate (SW)	SW1	II decomposition grade *Pinus massoniana* fallen log:the garden soil (1:1)
	SW2	II decomposition grade *Pinus massoniana* fallen log:granular soil (1:1)
	SW3	II decomposition grade *Pinus massoniana* fallen log:general-purpose nutrient soil (1:1)
	SW4	II decomposition grade *Pinus massoniana* fallen log:grass charcoal soil (1:1)
	SW5	III decomposition grade *Pinus massoniana* fallen log:the garden soil (1:1)
	SW6	III decomposition grade *Pinus massoniana* fallen log:granular soil (1:1)
	SW7	III decomposition grade *Pinus massoniana* fallen log:general-purpose nutrient soil (1:1)
	SW8	III decomposition grade *Pinus massoniana* fallen log:grass charcoal soil (1:1)
	SW9	IV decomposition grade *Pinus massoniana* fallen log:the garden soil (1:1)
	SW10	IV decomposition grade *Pinus massoniana* fallen log:granular soil (1:1)
	SW11	IV decomposition grade *Pinus massoniana* fallen log:general-purpose nutrient soil (1:1)
	SW12	IV decomposition grade *Pinus massoniana* fallen log:grass charcoal soil (1:1)

### Indicator measurement method

#### Cultivation substrate index determination

The physical and chemical property indexes of the substrate include bulk density, water holding capacity, total porosity, aeration porosity, water holding porosity, and the void ratio, which were measured before cultivation, drawing on the determination methods of Li et al.^34^ and Zhao et al.^35^. Nutrient property indicators include total nitrogen, total phosphorus, and total potassium. The solution to be measured was prepared by H_2_SO_4_-HCLO_4_ decoction method, the total nitrogen content was determined by CleverChem automatic interrupted chemical analyzer, the phosphorus content was determined by the molybdenum yellow colorimetric method, and the total potassium content was determined by the flame photometric method.

#### Determination of growth and physiological indexes of *P. acutum*

Lengthmeasurement: After 30 days of cultivation, ten mosses were randomly selected for each treatment every 10 days and their length was measured (accuracy 0.1 cm).

Coverage measurement: After 30 days of cultivation, take a picture every 10 days, and then measure the moss area with AutoCAD 2016 tracing, and the moss cover calculation formula was as follows.

$${\text{C}} = \left( {{\text{A}}/{\text{B}}} \right) \times 100\% .$$

Formula: C-moss coverage; A-moss area; B-planting area.

#### Determination of physiological indicators

Determination of chlorophyll content in *P. acutum* referred to the determination method of Bao et al.^36^, with slight modifications. After 120 days of growth in different cultivation substrates, the moss was taken out, washed and drained for later use, 0.2 g of new branches were weighed and put into a 10 ml centrifuge tube. Subsequently, 8 ml of 95% ethanol was added to the centrifuge tube. After 24 h of extraction under dark conditions, the liquid in the centrifuge tube and the moss residue were filtered several times and fixed into a 25 ml brown volumetric flask, and then the extract was poured into a 1 cm colorimetric cup of optical diameter. Finally, the absorbance at wavelengths of 665 nm and 649 nm was measured with a spectrophotometer with 95% ethanol as a blank control and then chlorophyll a content, chlorophyll b content, chlorophyll a/b, and total chlorophyll content was calculated according to the Arnon method. Soluble sugar content was determined by the anthrone colorimetric method, and soluble protein content was determined by the Komas Brilliant Blue G-250 (Bradford method).

#### A comprehensive evaluation of the growth and development status of the mosses

The fuzzy affiliation function evaluation method was used to comprehensively evaluate the growth and development status of the P.acutum. The calculation equation is as follows.

$${\text{F}}_{1} \left( {{\text{X}}_{{\text{i}}} } \right) = \left( {{\text{X}}_{{\text{i}}} - {\text{X}}_{\min } } \right)/\left( {{\text{X}}_{\max } - {\text{X}}_{\min } } \right),$$

$${\text{F}}_{2} \left( {{\text{X}}_{{\text{i}}} } \right) = \left( {{\text{X}}_{{\text{i}}} - {\text{X}}_{\min } } \right)/\left( {{\text{X}}_{\max } - {\text{X}}_{\min } } \right).$$

Formula: X denotes the measured value of the ith index, X_min_ denotes the measured minimum value of the ith index, and X_max_ denotes the maximum measured value of the ith index. Pearson correlation analysis was performed on the growth and physiological indexes of the *P. acutum*. If the indexes were opposite to the growth indexes of the *P. acutum*, the value of its affiliation function was calculated by using the inverse affiliation function formula F_2_. The values of the subordinate functions of the growth and development indexes of *P. acutum* under different cultivation substrates were accumulated and averaged to obtain the comprehensive evaluation index of plant morphology. The larger the index value is, the better the plant growth under the cultivation treatment of the substrate^37,38^.

### Statistical analysis

The data were analyzed by one-way ANOVA with Excel 2010 and SPSS19.0 software. Multiple comparisons were performed by Duncan’s (P < 0.05), and correlations between indicators were analyzed by Pearson correlation coefficients and plotted with Excel 2010 and Origin 2021.

### Licenses for collection of plant

The collection of plant material are comply with relevant institutional, national, and international guidelines and legislation.

## Data Availability

The datasets generated during or analysed during the current study are available from the corresponding author on reasonable request.
